# A decade on: the tailings legacy in Rio Doce water quality

**DOI:** 10.1007/s10661-026-15549-x

**Published:** 2026-06-20

**Authors:** Luciana Pena Mello Brandão, Bianca Loureiro do Valle, Renata Cristina Henedino Amancio, Winnícius Muniz dos Santos Sá, Arielli Giachini Zavaski, Estevão Emerick de Oliveira Eller, Leticia Malta Costa, José Fernandes Bezerra-Neto

**Affiliations:** 1 Limnea—Institute of Biological Sciences (ICB), Federal University of Minas Gerais (UFMG), Av. Antônio Carlos 6627, Pampulha, Belo Horizonte, Minas Gerais 31270-901 Brazil; 2 Departament of Chemistry, ICEX, Federal University of Minas Gerais (UFMG), Av. Antônio Carlos 6627, Pampulha, Belo Horizonte, Minas Gerais 31270-901 Brazil

**Keywords:** Long-term monitoring, Mining tailings, Rio Doce, Water pollution, Tropical rivers

## Abstract

In 2015, the collapse of the Fundão tailings dam released millions of cubic meters of mine waste into the Rio Doce Basin, causing profound and lasting environmental impacts. We analyzed 3 years of seasonal variation of water quality and metal concentrations across two middle Rio Doce sub-basins: one impacted by the tailings (Doce River) and a non-impacted reference system (Santo Antônio River). We hypothesized that tailings-associated variables (e.g., turbidity, Mn, As, Fe, Al) would differ in concentration and seasonality between the sub-basins, reflecting altered ecological responses nearly a decade after the collapse. Multivariate analyses (PCA, PERMANOVA) and mixed-effects models revealed significant spatial and temporal differences, with the impacted sub-basin showing persistently higher turbidity and metal loads, especially during the rainy season. Cluster analysis identified spatial groups corresponding to contamination gradients relative to the tailings source and confluence zones. Overall, our results demonstrate that the legacy of the tailings continues to shape water quality dynamics and suspended particulate matter in the Rio Doce system, highlighting the need for ongoing, spatially explicit monitoring to support ecosystem recovery and management.

**Supplementary Information:**

The online version contains supplementary material available at 10.1007/s10661-026-15549-x.

## Introduction

Mining is essential for supplying raw materials for industry and technological development. However, mining activities involve the large-scale mobilization of rocks and soils, intensive consumption of water and energy, and the generation of fine tailings that are stored in dams, resulting in multiple environmental impacts. When released into aquatic environments, tailings can amplify these impacts by altering sediment dynamics, contaminating water bodies, and compromising ecosystems for an indefinite period of time (Macklin et al., 2023). Among the most critical consequences of tailings dam operations are acid mine drainage and tailings releases, which substantially increase trace metals loads and suspended solids in watersheds worldwide (Singer and Stumm, 1970; Luo et al., 2020). Unlike other contamination sources, tailings dam failures are characterized by sudden, massive release of material that drastically alter geomorphology, sediment transport, and biotic communities (Macklin et al., 2006; Hatje et al., 2017).

The Doce River basin, located in southeastern Brazil, is a complex and historically degraded watershed that has long been subjected to substantial environmental pressures. These pressures stem from iron and gold mining, steel production, forestry, agriculture, livestock, urbanization, and inadequate sanitation infrastructure (Fernandes et al., 2016; Hatje et al., 2017).

This scenario was aggravated on November 5, 2015, by the collapse of the Fundão tailings dam operated by Samarco, BHP Billiton, and Vale in the district of Bento Rodrigues, municipality of Mariana (Minas Gerais State). The dam collapse released 43 million cubic meters of iron-ore tailings, severely compromising multiple water uses and aquatic ecosystems (ANA, 2016; Carmo et al., 2017; IGAM, 2017). This material, composed mainly of iron and silicon oxides, also contained high concentrations of Al, Fe, Mn and, in finer fractions, As, Cu, Cr, Hg, Ni, Pb, and Zn (Segura et al., 2016; Almeida et al., 2018). The mud flow traveled more than 668 km along the Gualaxo do Norte, Carmo, and Doce rivers to the Atlantic Ocean, causing sharp increases in turbidity (reported up to 606,200 NTU), burying riparian vegetation and benthic habitats, and producing mass mortality of aquatic organisms, directly impacting more than one million inhabitants across 41 municipalities (ANA, 2016).

Given the pre-existing anthropogenic pressures on the basin, isolating the chronic effects attributable to the dam collapse is challenging (Segura et al., 2016). In this context, comparative studies between impacted and unimpacted rivers are essential to detect parameters influenced by tailings releases, provide baselines, and understand long-term ecological recovery trajectories (Abushandi, 2025). Such data are fundamental for public-policy development, environmental management actions, and more effective restoration activities (Fernandes et al., 2016).

Besides the necessity of reference systems, seasonal variability is a major factor modulating water quality and contaminant dynamics in tropical basins such as the Doce River, which exhibit pronounced rainy and dry seasons (Passos et al. 2021; Garcia et al., 2024). During the rainy season, higher precipitation and discharge enhance erosion and resuspension processes that can remobilize tailings and sediments deposited on riverbed and banks (Banunle et al., 2024; de Souza Viana et al., 2024). This seasonal remobilization can markedly increase turbidity and suspended-solids concentrations and affect the dynamics and mobility of metals such as Al, Fe, and Mn (Santos et al., 2021). Understanding the influence of seasonality on water quality parameters is therefore essential to distinguish between natural variability and contamination legacies of the mining disaster.

In this context, we aimed to assess three years of seasonal variation in water quality in two sub-basins of the middle Doce River, one impacted by mine tailings (Doce River) and one unimpacted reference (Santo Antônio River), and to identify differences in parameters associated with the historical tailings impact. We hypothesized that concentrations and seasonal patterns of limnological variables and trace elements would differ between the two sub-basins, particularly for tailings-associated parameters (e.g., metals and turbidity). Such differences may reflect altered ecological response to the hydrological cycle, nearly a decade after the dam collapse.

## Materials and methods

### Study area

This study was conducted in the portion of the Doce River Basin located in Minas Gerais State, southeastern Brazil. Approximately 87% of the basin’s total area (86,700 km^2^) lies within Minas Gerais, encompassing 228 municipalities, 200 of which are in this state (ANA, 2013b; CBH-Doce, 2016a). The Doce River originates in the Mantiqueira and Espinhaço mountain ranges and flows approximately 850 km to the Atlantic Ocean in the Espírito Santo (ECOPLAN-Lume, 2010). The basin is characterized by a mosaic of land uses, with prominent activities including mining, agriculture, cattle ranching, reforestation, and steel and metallurgical industries; it hosts the largest steel-production complex in Latin America, closely linked to the mining and forestry sectors (CBH-Doce, 2016). The regional climate is tropical with two well-defined seasons: a rainy season from October to March (spring and summer), and a dry season from April to September (autumn and winter). Local climatic conditions were characterized using monthly rainfall and air temperature data from January 2022 to August 2025. A marked seasonal pattern was observed. During the rainy season (October–March), mean monthly rainfall was 204.6 ± 150.2 mm, whereas during the dry season (April–September), it averaged 33.8 ± 49.5 mm. Mean air temperature was also higher in the rainy season (24.6 ± 1.3 °C) compared to the dry season (21.1 ± 1.7 °C). These data confirm strong precipitation seasonality and moderate but consistent thermal variation in the study region. (INMET, 2025).

Two sub-basins were selected for the present study: the Doce River sub-basin and the Santo Antônio River sub-basin. The Doce River sub-basin includes areas directly impacted by the 2015 dam collapse, with significant environmental liabilities related to mining and intense anthropogenic land use. In contrast, the Santo Antônio River sub-basin, although part of the larger Doce River Basin, has lower population density, greater vegetation cover, and no direct influence from the mining tailings released during the Fundão dam failure, serving here as a reference area (CBH-Doce, 2016; ECOPLAN-Lume, 2010). The Santo Antônio River is one of the major tributaries of the Doce River.

### Sampling and analysis of water-quality parameters

**Fig. 1:**
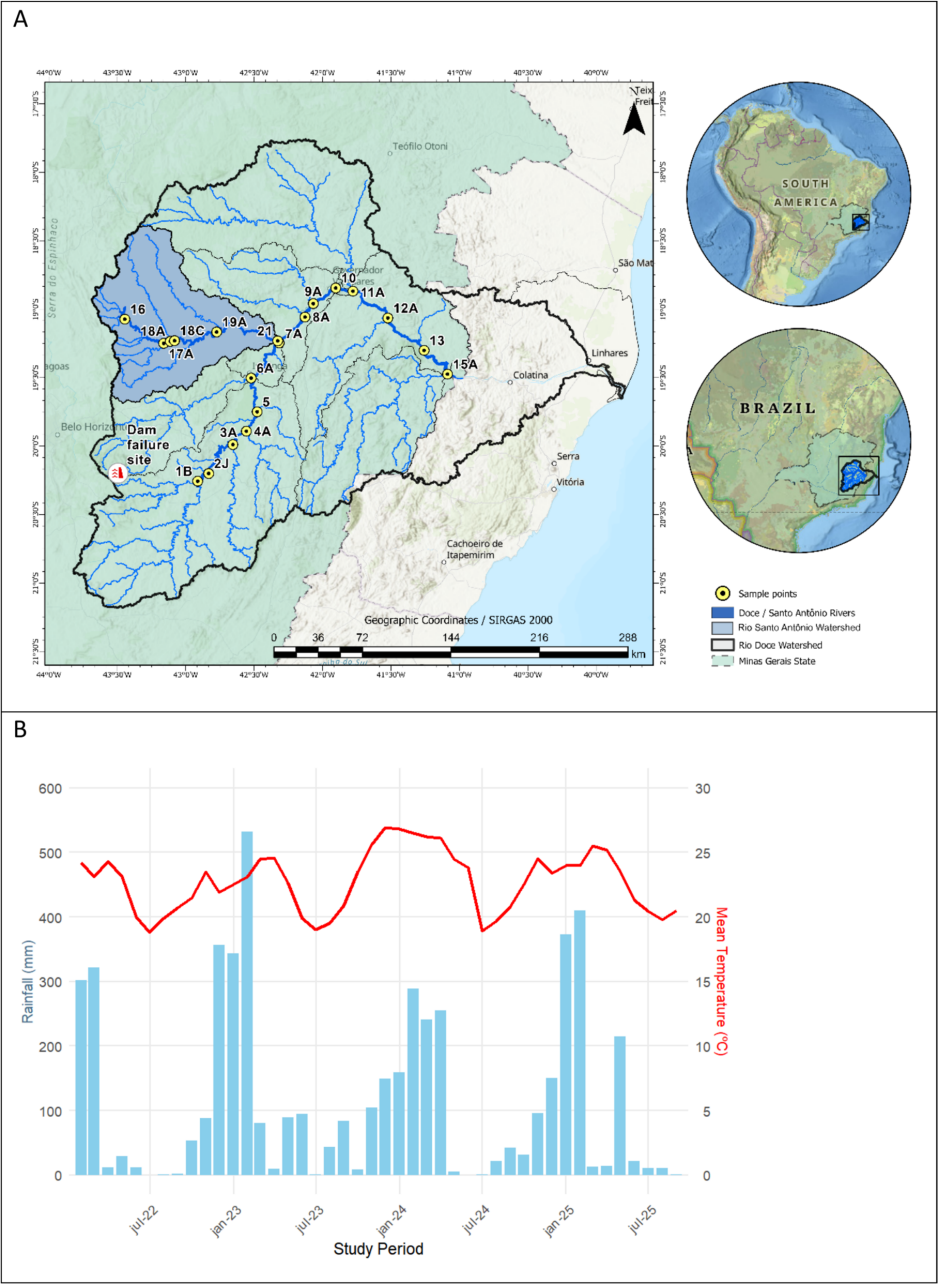
**A** Location of sampling sites in the middle Doce River Basin, highlighting the Doce (impacted) and Santo Antônio (reference) sub-basins and their confluence. **B** Monthly rainfall (mm) and mean temperature (^o^C) during the period (July 2022–July 2025), from the Timóteo meteorological station (INMET, station A511)

Sampling spanned 3e years and covered all four seasons: spring (October—rainy), summer (January—rainy), autumn (April—dry), and winter (July—dry) for a total of 11 field campaigns from July 2022 to January 2025. The sampling network comprised sites distributed along both the Doce (14 sites) and Santo Antônio (6 sites) rivers, located upstream and downstream of their confluence (Fig. 1). Sampling effort was intentionally unbalanced to reflect basin scale and study design: the larger, impacted mainstem (Doce River) was more densely sampled to capture spatial variability, whereas the smaller tributary (Santo Antônio River), included as a reference system, was represented by fewer sites sufficient to characterize baseline conditions.

Abiotic parameters were analyzed from triplicate water samples. Shortly after collection, water was filtered for chlorophyll-*a* (Chl-a) and total suspended solids (TSS, using GF/F 0.7 µm Whatman). Additional samples were filtered for dissolved organic carbon (DOC) using 0.22-μm Millipore filters and stored in amber glass bottles previously rinsed with distilled water and 10% hydrochloric acid. All DOC samples were kept at 4 °C in the dark until analysis. Filtrates were also collected for phosphate (μg L-1; 0.22-μm Millipore filter) and kept at 4 °C until analysis by ion chromatograph (Metrohm 883 Basic IC).

Chl-a (corrected for pheophytin; μg L^−1^) was determined after 90% acetone extraction, with absorbance readings taken at 665 and 750 nm on a UV–VIS spectrophotometer (Shimadzu, UV–Vis 2600), and concentrations were calculated according to APHA (1998). TSS (mg L^−1^) were determined gravimetrically as the difference between the dry weights of GF/F filters (105 °C for 1 h) before and after the filtration (APHA, 1998). DOC concentration (mg L^−1^) was measured by high-temperature catalytic oxidation on a TOC analyzer (Shimadzu TOC VCPN). Total nitrogen (TN, mg L^−1^) was quantified from unfiltered samples using a TOC analyzer (Shimadzu TOC VCPN). Water temperature, specific conductivity, pH, and dissolved oxygen were measured in situ with a YSI ProDSS multiparameter probe. Turbidity was measured using a Digimed turbidimeter, model DM-TU.

### Trace elements

Dissolved metals (Al, Cu, and Fe) were determined from water filtered through mixed cellulose ester (MCE) membranes with 0.45 μm pore size and preserved with the addition of one drop of 0.1 N HCl (APHA, 2017). In accordance with regulatory guidance and analytical constraints (CONAMA Resolution 357/2005) commonly applied in Brazilian water monitoring programs, only the dissolved fractions of Al, Cu, and Fe were analyzed, as regulatory thresholds are defined specifically for these metals in their dissolved form. These elements are naturally abundant in the region and occur predominantly in particulate-bound phases; therefore, total concentrations would largely reflect geological background rather than environmentally relevant contamination. The dissolved fraction, in turn, represents the potentially bioavailable pool, which is the most appropriate indicator for assessing ecological risk in this basin.

Total metals and metalloids concentrations (As, Cd, Cr, Mn, Ni, Pb, and Zn, in μg L^−1^) were obtained from unfiltered water samples, also preserved with 0.5% v/v nitric acid solution 0.1 N HCl. All samples were digested by microwave-acid-assisted digestion (Milestone Ethos 1 – Advanced Microwave Digestion System oven, Sorisole, Italy), performed in accordance with EPA Method 3051A. Subsequently, the samples were analyzed by inductively coupled plasma mass spectrometry (ICP-MS, série 7700, Agilent Technology) (APHA, 2017; Oliveira et al., 2025).

### Data analysis

Because of the marked hydrological seasonality in tropical regions, the general description of the water quality parameters is presented by grouping the sampling events into two climatic seasons: dry (autumn–winter) and rainy (spring–summer), and the significance of differences was tested using the Student’s *t*-test or the Mann–Whitney *U* test, depending on data normality. This approach provides a clearer overview of seasonal patterns. However, for the statistical analyses, each sampling campaign was considered as an independent temporal unit, as this level of resolution is necessary to evaluate differences in variability between the two sub-basins.

To evaluate the relative influence of spatial (sampling point) and temporal (sampling campaign) factors on water quality variables, we calculated the proportion of variance explained (R2) for each factor using linear models. Analyses were performed separately for each sub-basin and variable, including limnological parameters and trace elements concentrations. Boxplots were generated for the water quality parameters and trace elements, organized by sampling campaign and separated by sub-basin (Doce and Rio Santo Antônio) to visualize seasonal patterns and compare the distribution.

Principal component analysis (PCA) was applied to standardized environmental variables (*z*-scores) to explore multivariate patterns among sampling sites. Variables containing only missing values were excluded, and rows with incomplete data were removed. Outliers were defined as observations with absolute standardized values greater than 3 and were excluded. PCA was performed on the cleaned dataset using the prcomp() function in R. The first two principal components were visualized with biplots, including variable loadings and 95% confidence ellipses to highlight groupings by basin and seasons.

A permutation-based multivariate analysis of variance (PERMANOVA) was conducted using the adonis2 function from the vegan package in R to assess the effects of basin, campaign, and their interaction on the overall environmental fingerprint. Euclidean distances were calculated on *z*-score-standardized chemical and turbidity variables to construct the distance matrix. Terms were added sequentially (type = “reduced model”) and tested with 999 permutations.

To assess the spatial (between basins) and temporal (between campaigns) influence on environmental variables, Generalized Linear Mixed Models (GLMMs) were fitted using the *lmer* function from the lmerTest package, adopting a hierarchical structure, including sampling point as a random effect. The response variable was modeled as a function of the interaction between basin and campaign, both treated as fixed effects (response variable ~ basin x campaign + (1 | sampling point)). Here, “basin × campaign” refers to the statistical interaction between these two factors (i.e., whether differences between basins vary across the sampled campaigns, and does not exclusively represent seasonal variation, even though campaigns occurred in different seasons). Separate models were fitted for each environmental variable of interest (e.g., turbidity, pH, DO, metals). Type III Analysis of Variance (ANOVA) was used to test the significance of the fixed effects. For visualization, raw means and standard errors were calculated for each basin-campaign combination.

To identify patterns among the sampling points based on environmental variables, we conducted hierarchical cluster analysis. Variables were first selected and replicate measurements averaged for each location. Variables without variation were removed. Missing data were imputed using predictive mean matching (PMM) via the mice package, ensuring that the data structure remained consistent. A Yeo-Johnson transformation was then applied to normalize variable distributions, followed by standardization so that all variables contributed equally. Using the standardized data, we calculated a Euclidean distance matrix and performed hierarchical clustering with Ward’s method. The optimal number of clusters was evaluated using the average silhouette width, the Calinski–Harabasz index, and the gap statistic, considering solutions ranging from *k* = 2 to *k* = 6. These diagnostics indicated a tendency toward fewer groups (*k* = 2). However, a three-cluster solution (*k* = 3) was retained, as it better preserved ecologically meaningful differentiation among sites. The mean value of each variable within each cluster was plotted to identify the variables contributing most to group separation.

## Results

### General pattern and drivers of water quality in the sub-basins

The seasonal contrasts among the water quality parameters and trace elements concentrations are detailed in Table 1. Overall, trace elements concentrations, suspended solids, and turbidity were consistently higher in the Doce River sub-basin during both seasons. During the rainy season, mean turbidity, TSS, and key trace elements were higher in the Doce River compared to the Santo Antônio River (turbidity, Doce 218.14 vs. Santo Antônio 153.55 NTU; TSS, 189.01 vs. 113.64 mg L^−1^; Mn, 199.70 vs. 82.17 µg L^−1^; As, 2.51 vs. 0.32 µg L^−1^; Pb, 6.06 vs. 4.83 µg L^−1^; Ni, 19.85 vs. 8.84 µg L^−1^; Cr, 10.23 vs. 2.69 µg L^−1^; Zn, 12.99 vs. 11.69 µg L^−1^). In contrast, dissolved Fe was overall higher in the Santo Antônio River during rainy seasons (rain, 301.57 vs. 276.14 µg L^−1^; dry, 185.08 vs. 191.02 µg L^−1^), with only slightly higher values in the Doce River during the dry period (Table 1).

**Table 1:** Mean values and standard deviations of physicochemical parameters for each sub-basin, grouped by climatic season. Rainy season values correspond to the mean of sampling campaigns conducted in spring (October) and summer (January), whereas dry season values correspond to the mean of campaigns conducted in autumn (April) and winter (July). *P*-values (dry vs. rainy) were obtained using Student’s *t*-test or Mann–Whitney *U* test, depending on data normality

	**Doce**			**Santo Antônio**		
**Parameter**	**Rainy**	**Dry**	***p*-value**	**Rainy**	**Dry**	***p*-value**
Chla (µg/L)	3.27 ± 7.09	0.31 ± 0.85	**0.010**	4.04 ± 11.94	1.48 ± 2.23	0.822
TSS (mg/L)	189.01 ± 297.18	108.76 ± 174.79	**0.001**	113.64 ± 182.55	78.66 ± 178.47	**0.020**
Phosphate (µg/L)	18.75 ± 39.47	27.77 ± 59.12	0.305	35.13 ± 45.69	36.49 ± 67.29	0.713
DOC (mg/L)	4.62 ± 3.32	8.12 ± 5.23	** < 0.001**	6.18 ± 4.67	14.80 ± 13.11	** < 0.001**
TN (mg/L)	2.83 ± 2.57	1.63 ± 1.91	0.270	1.45 ± 1.69	0.84 ± 0.80	0.504
Temperature (°C)	27.43 ± 1.19	24.74 ± 2.71	** < 0.001**	25.51 ± 1.47	22.69 ± 2.71	** < 0.001**
pH	7.30 ± 0.46	7.32 ± 0.48	0.273	7.35 ± 0.57	7.18 ± 0.76	0.105
OD (mg/L)	8.52 ± 0.55	9.38 ± 0.68	** < 0.001**	8.93 ± 0.60	9.42 ± 0.90	**0.022**
Conductivity (µS/cm)	75.75 ± 26.16	99.38 ± 35.28	** < 0.001**	38.93 ± 26.00	74.98 ± 73.94	**0.009**
Turbidity (NTU)	218.14 ± 238.14	37.83 ± 47.33	** < 0.001**	153.55 ± 235.56	18.00 ± 32.07	** < 0.001**
Al (diss) (µg/L)	39.45 ± 46.25	32.89 ± 43.85	0.538	45.63 ± 53.51	28.56 ± 49.75	0.109
Fe (diss) (µg/L)	276.14 ± 181.31	191.02 ± 115.02	**0.002**	301.57 ± 96.92	185.08 ± 65.08	** < 0.001**
Cu (diss) (µg/L)	0.93 ± 1.43	0.54 ± 1.16	**0.021**	0.76 ± 1.15	1.19 ± 4.25	0.491
Mn (µg/L)	199.70 ± 175.92	56.55 ± 41.88	** < 0.001**	82.17 ± 61.27	62.79 ± 87.67	**0.003**
As (µg/L)	2.51 ± 2.40	0.54 ± 0.43	** < 0.001**	0.32 ± 0.38	0.15 ± 0.41	**0.001**
Pb (µg/L)	6.06 ± 7.33	0.90 ± 1.16	** < 0.001**	4.83 ± 9.32	0.62 ± 0.91	** < 0.001**
Cd (µg/L)	0.01 ± 0.03	0.01 ± 0.06	0.062	0.01 ± 0.04	0.07 ± 0.33	0.793
Zn (µg/L)	12.99 ± 16.68	5.42 ± 9.60	**0.003**	11.69 ± 21.02	13.53 ± 18.39	0.344
Cr (µg/L)	10.23 ± 18.52	1.82 ± 3.51	0.130	2.69 ± 5.15	4.87 ± 16.92	0.281
Ni (µg/L)	19.85 ± 32.05	1.53 ± 3.93	** < 0.001**	8.84 ± 18.68	1.60 ± 4.53	**0.026**

A similar pattern was observed during the dry season, when the Doce River also showed higher values for most parameters (turbidity, 37.83 vs. 18.00 NTU; TSS, 108.76 vs. 78.66 mg L^−1^; Mn, 56.55 vs. 62.79 µg L^−1^—slightly higher in Santo Antônio; As, 0.54 vs. 0.15 µg L^−1^; Pb, 0.90 vs. 0.62 µg L^−1^; Ni, 1.53 vs. 1.60 µg L^−1^—similar between basins; Cr, 1.82 vs. 4.87 µg L^−1^—higher in Santo Antônio) (Table 1).

Regarding the other water quality parameters, the Doce River generally exhibited higher concentrations of nutrients and organic matter during the rainy season, with elevated chlorophyll-a, DOC, and TN compared to the Santo Antônio River. In contrast, during the dry season, DOC and TN were notably higher in the Santo Antônio sub-basin. Physical parameters also showed distinct patterns: water temperature was consistently higher in the Doce River across both seasons, while pH values were similar between basins, with only slight differences. Dissolved oxygen showed minimal variation, with slightly higher values in the Santo Antônio River during the dry period. Conductivity, however, was markedly higher in the Doce River in both seasons, reflecting the stronger influence of suspended solids and dissolved ions in this sub-basin. These contrasts highlight consistent physicochemical differences between the two systems throughout the monitoring period (Table 1).

**Fig. 2:**
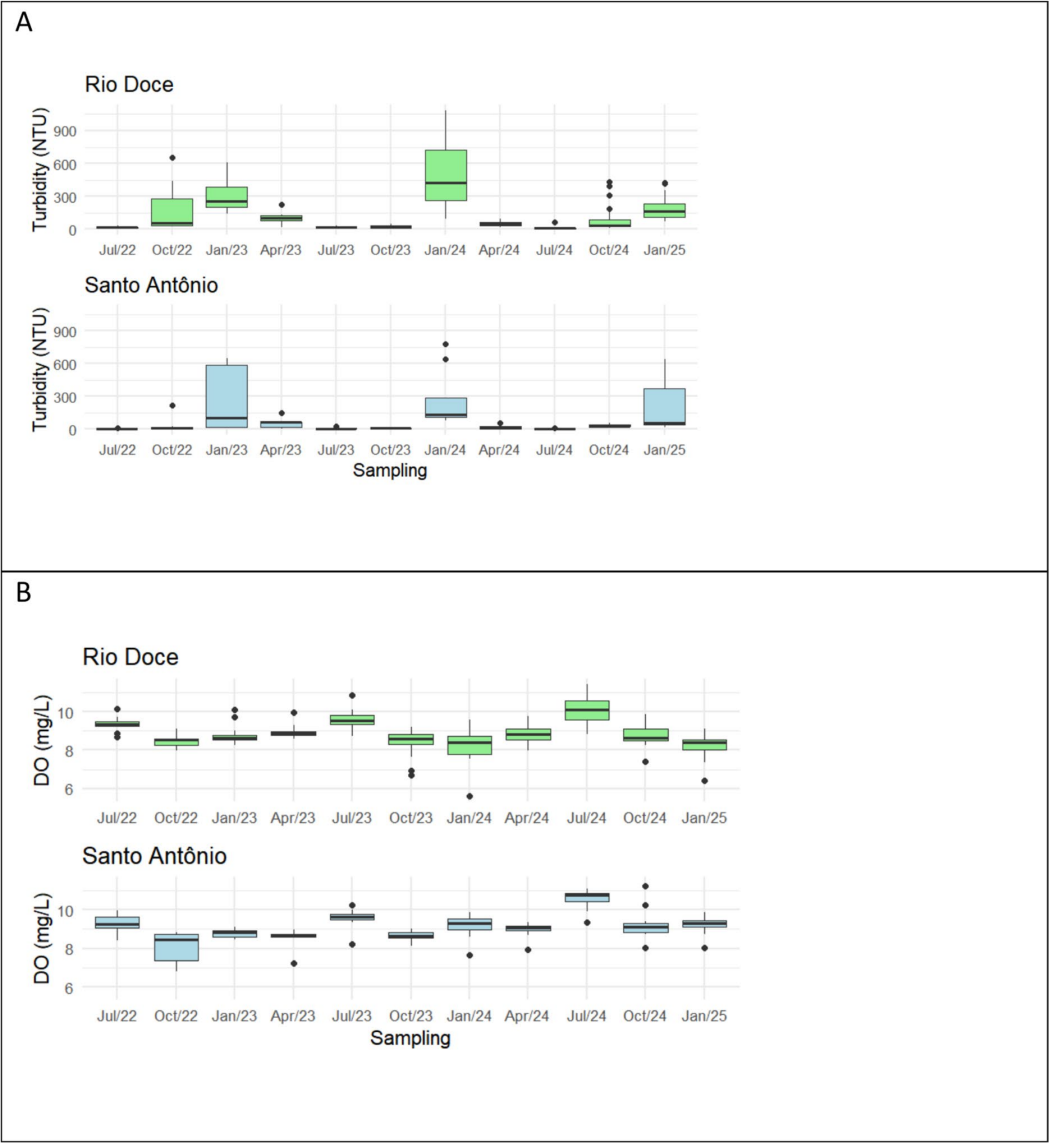
Temporal variation of limnological parameters across sampling campaigns in the Doce and Santo Antônio rivers. Boxplots show the following: **A** turbidity (NTU), **B** dissolved oxygen-DO (mg L^−1^). Boxes indicate the interquartile range (25–75th percentile), the horizontal line is the median, whiskers extending to 1.5 × the interquartile range, and points denote outliers

In Fig. 2, we highlight the seasonal pattern of turbidity in both sub-basins, with higher values during the rainy season, particularly in summer (January), and consistently greater levels in the Doce River. In contrast, higher dissolved oxygen concentrations occur during the dry winter period (July).

**Fig. 3:**
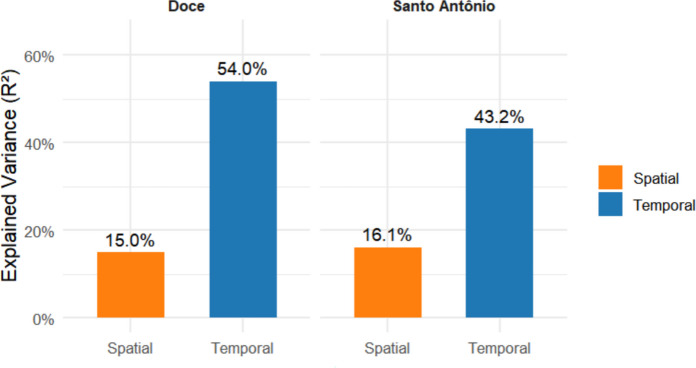
Variance partitioning of water quality variables by campaign (temporal) and sampling point (spatial) for each sub-basin. Bars show the proportion of variance explained (R2) by each factor

Across both sub-basins, limnological parameters and trace elements concentrations exhibited a markedly stronger temporal than spatial effect (Fig. 3). Variance partitioning revealed that sampling campaigns consistently explained a higher proportion of variance (R2) than sampling points, indicating that temporal dynamics were the primary drivers of water quality variability. This pattern held across all variables analyzed (Fig. S1 – Electronic Supplementary Material).

### Multivariate patterns between sub-basins

PERMANOVA on Euclidean distances of *z*-score-standardized turbidity and both total and dissolved metal concentrations revealed significant main effects of basin (Df = 1, R2 = 0.0298, F = 5.80, *p* = 0.001) and campaign (Df = 9, R2 = 0.3535, F = 7.66, *p* = 0.001), and a significant basin × campaign interaction (Df = 8, R2 = 0.0680, F = 1.66, *p* = 0.004). These results indicate distinct temporal trajectories of water quality parameters in the Doce and Santo Antônio sub-basins.

**Fig. 4:**
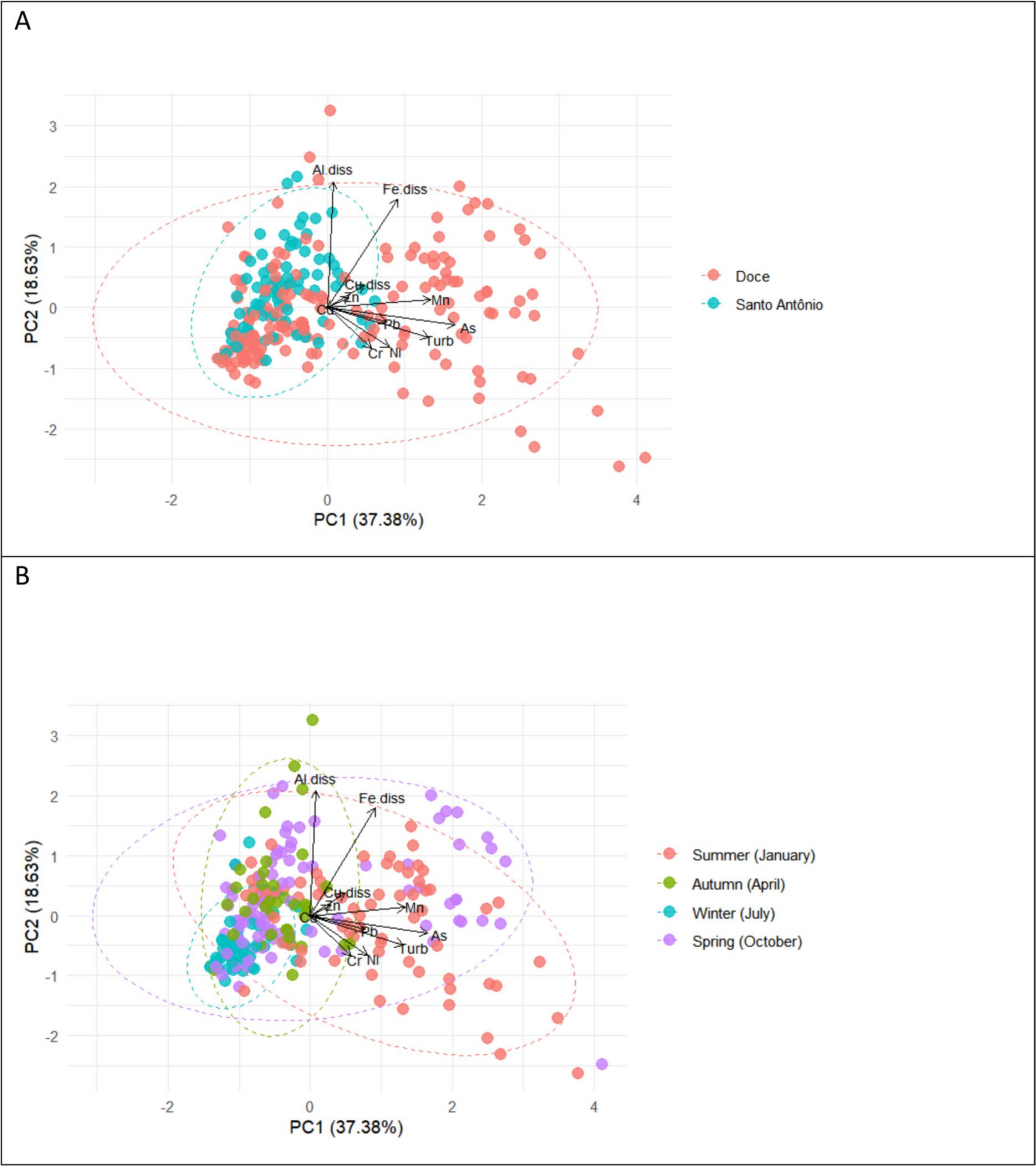
Principal component analysis (PCA) of standardized variables. **A** Scores with 95% confidence ellipses by sub-basin (Doce vs. Santo Antônio). **B** Scores with 95% confidence ellipses by season (rainy: Oct/Jan; dry: Apr/Jul). Variables used are as follows: turbidity (Turb; NTU), dissolved aluminum (Al diss; µg L^−1^), dissolved iron (Fe diss; µg L^−1^), dissolved copper (Cu diss; µg L^−1^), and total trace elements Mn, As, Pb, Cd, Zn, Cr, Ni (µg L^−1^). Loadings are shown as vectors; axes display variance explained (%)

The variation of parameters associated with the tailings (turbidity and trace elements) between sub-basins and among seasons is shown in Fig. 4. A partial separation between sub-basins was observed along the PC1, indicating differences in the environmental profiles. Doce samples showed greater dispersion and tended to be associated with higher concentrations of trace elements such as Mn, As, Ni, Pb, Cr, and Fe, and with higher turbidity. On the other hand, Santo Antônio samples showed a more concentrated distribution, indicating lower variability and concentrations for most parameters. Dissolved Al was high in both sub-basins (Fig. 4 A). An additional PCA performed using the cluster-derived spatial groups (Fig. S2) further clarified that the Doce River samples overlapping with the Santo Antônio River samples in Fig. 4 A correspond to sites located downstream of the confluence between the Santo Antônio and Doce rivers, reinforcing the longitudinal dilution effect along the river continuum.

Higher turbidity and increased concentrations of total and dissolved metals were also observed during the summer and autumn (rainy season) (Fig. 4B).

Generalized linear mixed models (GLMMs) were fitted to evaluate the effects of basin, campaign, and their interaction on the physicochemical variables and trace elements (Table 2). Most environmental variables exhibited a significant temporal effect. Among the physicochemical variables, DOC, total nitrogen (TN), temperature, specific conductivity, and turbidity showed significant basin effects. Additionally, DOC, TN, and OD showed significant interaction effects between basin and campaign (basin × campaign), indicating that the temporal patterns differed between the two sub-basins. Additionally, DOC, TN, and OD showed significant basin x campaign interactions.

Within the set of trace elements, As and Mn showed significant effects of both basin and its interaction with campaign. Other metals, such as dissolved Fe, Cd, and Zn, exhibited significant basin x campaign interaction effects. Ni showed a significant basin effect but no significant interaction. For the remaining variables, basin, campaign, and interaction effects were not statistically significant.

**Fig. 5:**
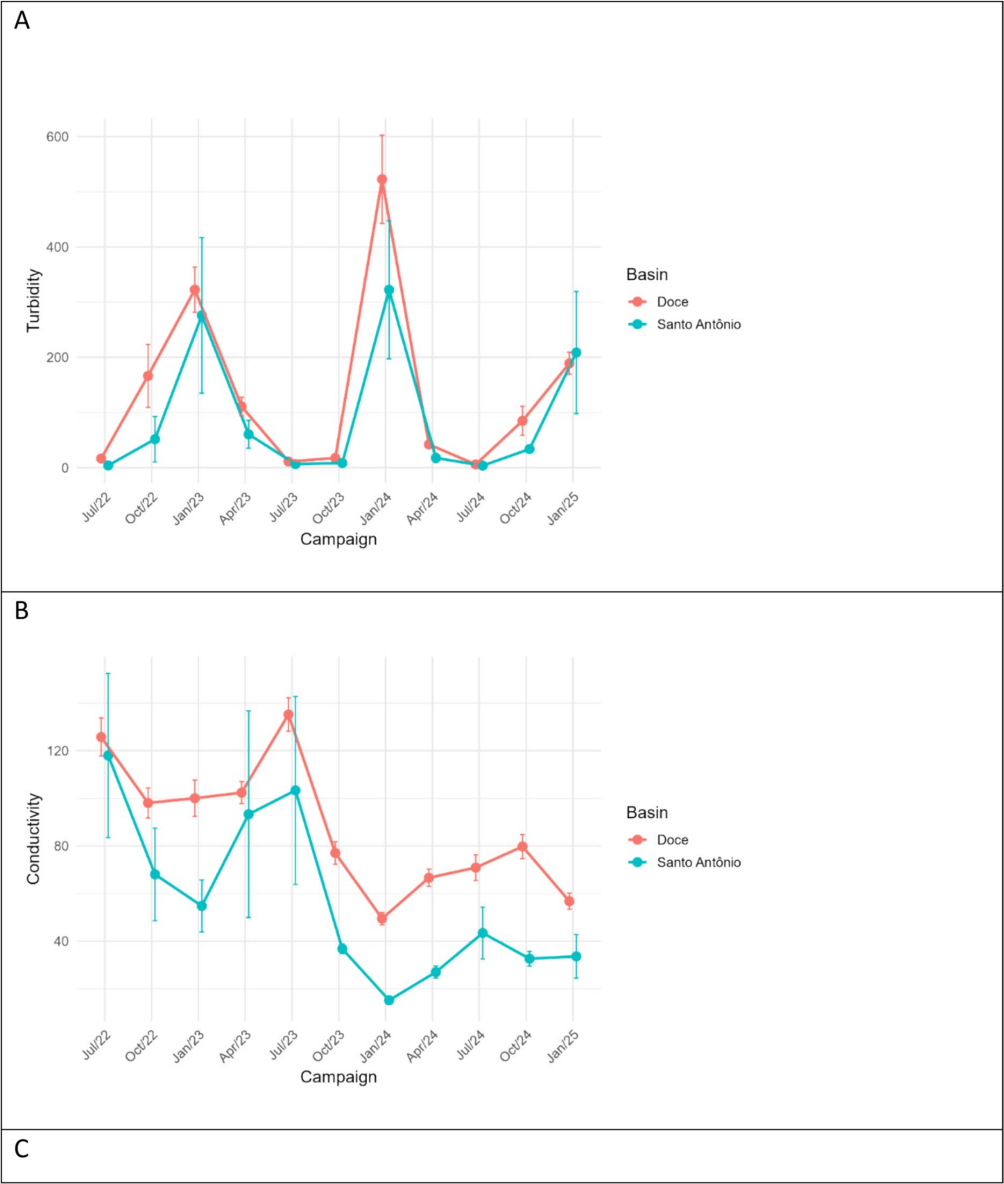

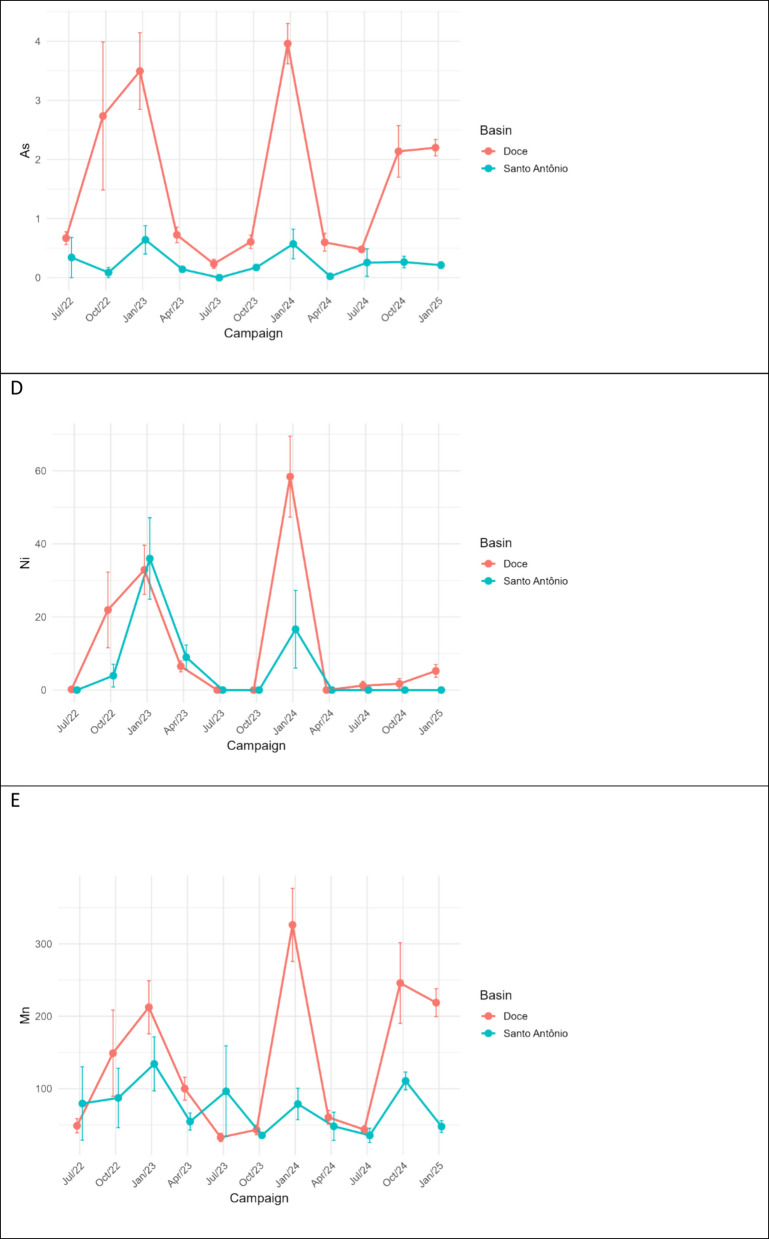

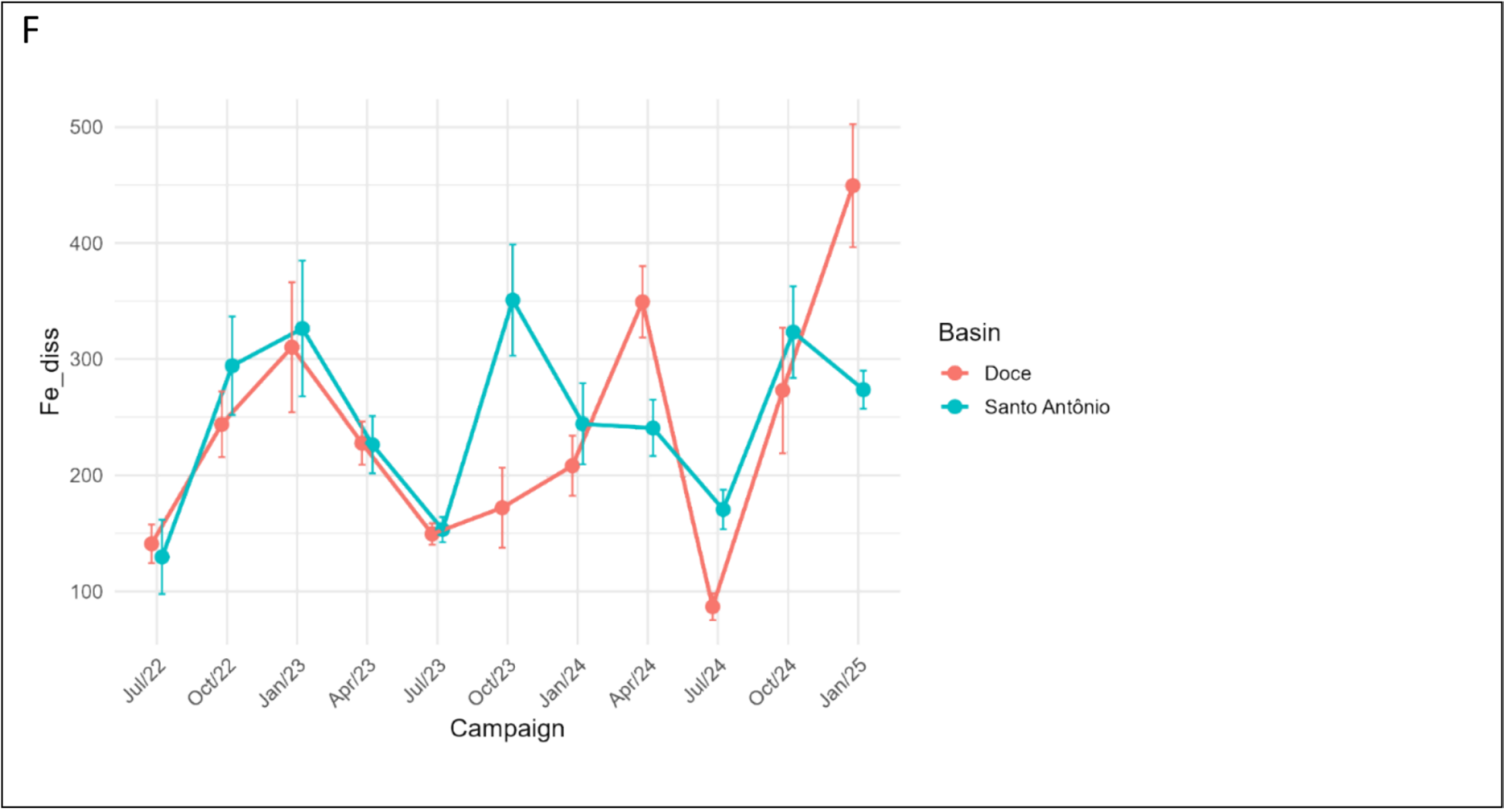
Effects of sub-basin, campaign, and their interaction on physicochemical variables and trace elements concentrations from generalized linear mixed models (GLMMs). Panels show predicted means ± standard errors for the following: **A** turbidity (NTU), **B** specific conductivity (µS cm^−1^), **C** As (µg L^−1^), **D** Ni (µg L^−1^), **E** Mn (µg L^−1^), and **F** dissolved Fe (µg L^−1^)

The mixed-effects models revealed a consistent seasonal pattern for some parameters, including turbidity, As, Ni, Mn, and dissolved Fe, with increases during the rainy season ((spring—October and summer—January) (Fig. 5 A, C, D, E, F). The analysis also showed a significant temporal effect on specific conductivity (*p* < 0.05), with a progressive decrease across the monitoring period in both basins (Fig. 5B). Notably, these parameters, potentially linked to tailings contamination, consistently exhibited higher concentrations in the Doce River sub-basin throughout the study.

Table 2 Results of Type III ANOVA from Generalized Linear Mixed Models (GLMMs) assessing the effects of basin, campaign, and their interaction on selected environmental variables. Only statistically significant results are shown (*p* < 0.05). Significance levels are indicated as follows: *p* < 0.05 (*), *p* < 0.01 (**), and *p* < 0.001 (***)

**Table 2:** Results of Type III ANOVA from Generalized Linear Mixed Models (GLMMs) assessing the effects of basin, campaign, and their interaction on selected environmental variables. Only statistically significant results are shown (p < 0.05). Significance levels are indicated as follows: p < 0.05 (*), p < 0.01 (**), and p < 0.001 (***)

Parameter	Effect	df1	df2	*F*	*p*-value significance
Chla	Campaign	10	200.000	9.753	***
TSS	Campaign	10	196.000	5.692	***
Phosphate	Campaign	9	112.803	4.980	***
DOC	Basin	1	29.045	12.081	**
DOC	Campaign	10	140.770	24.718	***
DOC	Basin:campaign	10	140.770	9.773	***
TN	Basin	1	30.592	12.052	**
TN	Campaign	10	132.643	39.837	***
TN	Basin:campaign	10	132.643	5.008	***
Temp	Basin	1	18.119	14.862	**
Temp	Campaign	10	169.379	48.478	***
pH	Campaign	10	197.000	4.334	***
OD	Campaign	10	168.847	22.723	***
OD	Basin:campaign	10	168.847	3.406	***
Conductivity	Basin	1	18.353	11.637	**
Conductivity	Campaign	10	169.723	20.749	***
Turbidity	Basin	1	181.000	4.542	*
Turbidity	Campaign	10	181.000	16.171	***
Al_diss	Campaign	10	171.179	11.265	***
Fe_diss	Campaign	10	170.461	7.988	***
Fe_diss	Basin:campaign	10	170.461	3.008	**
Cu_diss	Campaign	10	173.297	6.641	***
Mn	Basin	1	18.308	5.194	*
Mn	Campaign	10	170.033	5.411	***
Mn	Basin:campaign	10	170.033	3.153	***
As	Basin	1	18.946	14.650	**
As	Campaign	10	171.480	4.972	***
As	Basin:campaign	10	171.480	3.220	***
Pb	Campaign	10	172.093	11.733	***
Cd	Campaign	10	172.960	2.674	**
Cd	Basin:campaign	10	172.960	2.919	**
Zn	Campaign	10	172.884	5.573	***
Zn	Basin:campaign	10	172.884	2.549	**
Cr	Campaign	10	198.000	9.629	***
Ni	Basin	1	198.000	4.229	*
Ni	Campaign	10	198.000	16.283	***

### Spatial patterns of water quality

**Fig. 6:**
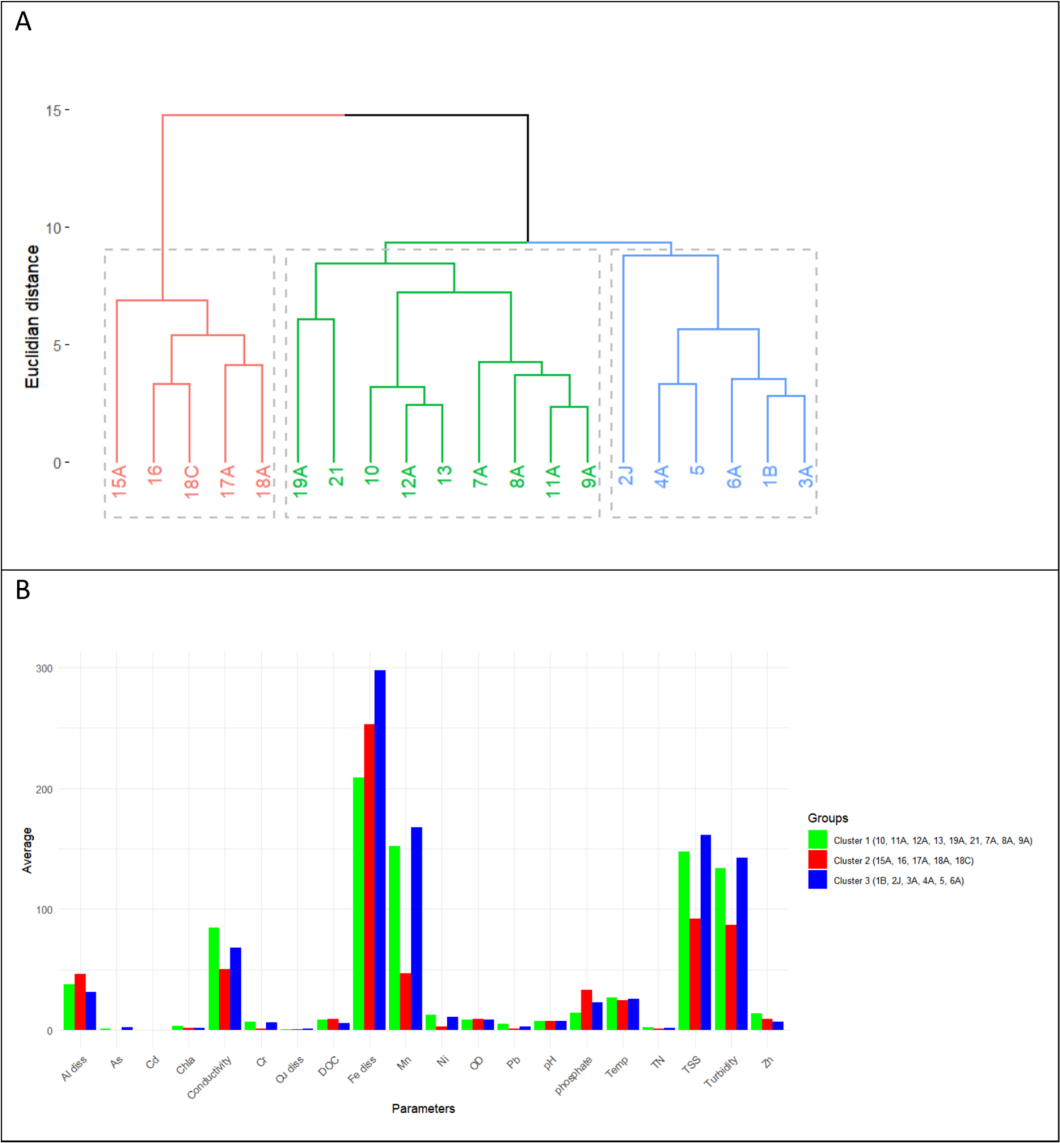
**A** Dendrogram from hierarchical clustering analysis (Ward’s method; Euclidean distance) based on standardized limnological variables and trace elements. **B** Cluster-wise mean concentrations of selected variables, highlighting distinct environmental profiles among the sampling sites. Clusters are labeled 1–3 as shown in **B**

Considering all the parameters measured in this study, cluster analysis revealed a clear separation among groups. Three distinct clusters reflected the spatial distribution of sampling points: one comprising mostly Santo Antônio sites (group Red), and two distinguishing upstream (Blue) and downstream (Green) reaches of the Doce River relative to its confluence with the Santo Antônio (Fig. 6 A). Figure 6 B illustrates the mean concentrations of each analyzed variable within each cluster group. For transparency and completeness, descriptive statistics for each cluster group are provided in the Supplementary Material (Table S2).

The Red Group comprised primarily sites from Santo Antônio (points 16, 18 C, 17 A, and 18 A) and was characterized by the highest mean concentrations of dissolved Al and phosphate. Turbidity, total suspended solids (TSS), and conductivity were lower compared to the other two cluster groups. These sites also showed elevated mean concentrations of dissolved Fe, although to a lesser extent than those observed in the Blue Group. Overall, dissolved Fe concentrations in the Red Group were lower than in the Blue Group but higher than in the remaining group.

Group Blue comprised all Doce sites upstream of the confluence (points 2 J, 4 A, 5, 6 A, 1B, 3 A) and was characterized by the highest dissolved Fe and total Mn, and by the highest turbidity and TSS among groups.

The Green Group comprised mainly Doce River sites located downstream of the confluence (points 10, 12 A, 13, 7 A, 8 A, 11 A, and 9 A), as well as two sites from the Santo Antônio River (points 19 A and 21), which are the closest to the confluence with the Doce River. These sites from this group were distinguished by the highest mean values of specific conductivity. They also exhibited high turbidity, TSS, and total Mn concentrations, although these were lower than those observed in the Blue Group. Despite presenting relatively elevated mean dissolved Fe concentrations, this group showed the lowest values among the three clusters. Additionally, the Green Group displayed the lowest phosphate concentrations of all groups analyzed.

## Discussion

The results of this long-term monitoring effort highlight persistent alterations in water quality within the Doce River sub-basin, nearly a decade after the Fundão tailings-dam collapse. A key finding was that temporal variation consistently explained a greater proportion of the variance in limnological parameters and trace elements than spatial variation. Hydrological analyses of the Rio Doce basin by Arndt et al. (2022) demonstrated that river discharge is strongly controlled by the seasonal rainfall regime, with higher flows and sediment inputs during the rainy season and markedly reduced discharge in the dry months. These shifts in flow directly influence suspended-sediment concentrations and metal transport, indicating that hydrological variability governs the magnitude and timing of material fluxes across the system. Interannual fluctuations in precipitation further modulate these patterns, reinforcing the dominant influence of temporal drivers on water quality dynamics. This aligns with our results and reinforces that seasonal and interannual hydrological changes, typical of tropical basins such as the Doce, are stronger drivers of water quality and contaminant dynamics than local spatial heterogeneity (Passos et al. 2021).

Despite the strong seasonal signal, it was possible to identify differences between the sub-basins. Consistently higher turbidity, TSS, and trace elements concentrations (Mn, As, Pb, Ni, Cr, Zn) in the Doce River sub-basin throughout the monitoring period (Table 1) are indicative of a lasting influence of tailings deposits. These parameters are commonly associated with mining residues and sediment resuspension (Richard et al. 2020), as well as with the composition of the mining waste from the Fundão dam (Segura et al. 2016; Almeida et al. 2018), supporting the hypothesis of a persistent impact nearly a decade after the collapse. The rainy-season increase in turbidity and trace elements levels observed further support this, reflecting intensified surface runoff and sediment transport typical of tropical watersheds under high precipitation regimes (Banunle et al., 2024). Similar seasonal patterns were reported by Quaresma et al. (2022) in the Parauapebas River Basin, southeastern Amazon, where higher concentrations of turbidity, iron, manganese, and aluminum were consistently observed during the rainy season, emphasizing the role of hydrological seasonality in mobilizing contaminants in mining-influenced tropical catchments.

Although both sub‑basins exhibited similar seasonal patterns characterized by increased turbidity and TSS during the rainy periods, the magnitude of these increases was significantly higher in the Doce River. This pattern suggests an altered hydro‑sedimentological regime in the Doce basin, likely driven by legacy contamination from tailings, as inferred from water-column parameters. Elevated concentrations of trace elements typically associated with mining activities (such as Mn, As, and Pb) during the rainy season in the Doce sub‑basin further support this interpretation, highlighting the ongoing influence of the disaster on trace elements mobilization and exposure routes (de Matos et al. 2022). The higher dissolved-Fe values observed in the Santo Antônio sub-basin in some campaigns (Fig. 5 F) therefore likely reflect local geochemical and hydrological drivers, such as geological background, groundwater inputs, and redox-mediated release, rather than particulate transport or direct influence of the tailings. This distinction highlights that while dissolved Fe behaves differently across sub-basins, particulate-associated metals continue to show clearer signatures of rainy-season mobilization in the Doce River sub-basin.

Conversely, phosphate, DOC, and specific conductivity showed higher values during dry periods in both basins, likely due to reduced streamflow, which limits dilution and increases solute concentrations at lower water levels (Tank et al., 2010). Elevated Cu, Zn, and Cr in the Santo Antônio sub-basin during the dry season may reflect local geochemical or land-use influences (Pimenta, 2021).

The multivariate analysis provided important insights into the factors shaping water quality in the study area. PERMANOVA results revealed significant effects of basin, season, and their interaction on turbidity and trace elements concentrations. Although the interaction effect size explained a small proportion of the variance (~ 6.8%), this significance suggests subtle but ecologically relevant seasonal differences in water quality dynamics between sub-basins. This relatively low proportion of explained variance is common in complex environmental datasets, where multiple factors and natural variability influence the measured parameters (Yigit, 2025). Taken together, these results highlight consistent seasonal differences in responses between the two sub-basins, reflecting subtle but ecologically meaningful patterns in turbidity and trace elements concentrations. In this context, PCA results showed that Doce River samples exhibited greater variability and were associated with higher concentrations of trace elements (dissolved Fe, dissolved Al, dissolved Cu, Mn and As) and turbidity. By contrast, Santo Antônio samples were more homogeneous and less associated with these parameters, reflecting a more stable and typical seasonal response to hydrological cycles.

Generalized linear mixed models identified significant temporal effects for most variables and confirmed basin-specific differences and basin:time interactions for key parameters. Among physicochemical variables, DOC, TN, turbidity, temperature, and specific conductivity showed significant basin effects. Additionally, DOC, TN, and dissolved oxygen showed significant interaction effects between basin and campaign, suggesting altered limnological functioning in the Doce River basin. Turbidity, a direct indicator of suspended particles and strongly associated with tailings input into the river (Richard et al. 2020), was significantly higher in the impacted basin throughout the monitoring period. Arsenic (As) was the only trace element showing both significant basin and interaction effects, indicating distinct seasonal patterns. Dissolved Fe, Mn, Cd, and Zn also displayed significant temporal dynamics differing by basin, while Ni showed significant basin differences without interaction effects. These findings point to persistent altered biogeochemical dynamics linked to the tailings’ legacy (Richard et al. 2020; Kütter et al. 2023).

It is important to highlight that the Santo Antônio River is a significant tributary of the Doce River and that the comparison between the sub-basins included sampling points both upstream and downstream of their confluence. Although this basin-level approach was aligned with our original hypothesis (impacted vs. reference sub-basin), we acknowledge that it may reduce the resolution of local spatial gradients within the Doce River. Furthermore, the influence of the Santo Antônio River on the water quality of the Doce River downstream of the confluence may contribute to an improvement in water quality in this section.

Nevertheless, even considering the potential dilution or modification effects caused by the inflow of the Santo Antônio River, the two sub-basins remained clearly distinct in their limnological characteristics throughout the study period. This persistent differentiation reinforces the interpretation that the Doce River still reflects the long-term legacy of the tailings impact, nearly a decade after the dam collapse.

In this context, cluster analysis revealed three distinct spatial groups: the Santo Antônio River, the Doce River upstream of the confluence with Santo Antônio, and the downstream confluence zone. This emergent spatial structuring supports the ecological relevance of segment-level heterogeneity within the basin.

The points located in the Santo Antônio River formed a distinct group, with a distinct chemical signature. It was characterized by the highest phosphate and dissolved Al concentrations, but lower turbidity, TSS, and conductivity compared to the Doce River groups. These results indicate comparatively lower particulate load but greater nutrient enrichment, reflecting natural differences in geology, land use, or catchment characteristics rather than the influence of the tailings.

The upstream Doce group, closest to the tailings source, included points with the highest turbidity, suspended solids, and metal concentrations (notably Mn and Fe), suggesting greater sediment input and metal mobilization near the original disaster site.

The downstream Doce group showed intermediate conditions. Although turbidity, TSS, and total Mn remained relatively high, they were lower than in the upstream Doce group. This downstream group was distinguished by the highest specific conductivity, indicating greater ionic content, possibly reflecting cumulative upstream inputs and mixing processes. Dissolved Fe and phosphate concentrations were the lowest among clusters groups, suggesting dilution or geochemical attenuation processes downstream of the confluence.

Interestingly, downstream of the confluence, the water quality profile appears to shift, with a general dilution of turbidity and metals such as Mn. Richard et al. (2020) also observed spatial differences along the basin regarding the effects of the Fundão dam breach on water quality, with impacts being more intense in areas closer to the dam. They further reported that these effects gradually diminished over time.

However, our results suggest that a persistent long-term impact remains in the Doce River basin. This spatial heterogeneity in water quality reflects the enduring imprint of the tailings on the Doce River’s limnology, modulated by hydrological connectivity and biogeochemical processes at the confluence zones. Importantly, the persistence of basin-level differences despite partial downstream dilution indicates that combining upstream and downstream Doce sites did not obscure the primary impacted vs. reference contrast that underpinned our study design. The fact that the Doce and Santo Antônio sub-basins remain limnologically distinguishable despite their hydrological connectivity highlights the resilience of the reference system and underscores the sustained imprint of the tailings on the Doce River. The persistence of altered water quality patterns across space and time highlights complex post-disaster ecological dynamics.

The sustained differences in water quality dynamics between the impacted and non-impacted sub-basins nearly ten years after the Fundão dam collapse highlight the persistence of ecological alteration in the Doce River system. These differences are evident not only in baseline concentrations but also in seasonal responses to hydrological variability, suggesting modified ecosystem functioning. However, it is important to recognize that the Santo Antônio River represents a spatial control rather than a true historical baseline, and that the Doce River Basin has long been subject to anthropogenic pressures. Historical monitoring data prior to the Fundão dam collapse indicate that portions of the basin already exhibited moderate turbidity, nutrient enrichment, and metal concentrations (Garcia et al. 2024). Thus, the patterns observed here likely reflect the persistence and amplification of pre-existing impacts combined with the legacy of the tailings. The results reinforce the importance of continued long-term, spatially explicit monitoring to detect subtle but ongoing impacts, support ecological risk assessments, and inform adaptive watershed management strategies. Understanding how hydrological connectivity regulates contaminant redistribution and ecological responses is essential for promoting recovery and mitigating risks in post-impact riverine systems (Dwivedi et al. 2025).

## Conclusion

Nearly 10 years after the Fundão dam collapse, our 3-year monitoring study demonstrates that the Doce River sub-basin still exhibits persistent alterations in water quality. Consistently higher turbidity, suspended solids, and tailings-associated trace elements (e.g., Mn, As, Pb, Ni, Cr, Zn), along with distinct seasonal responses, confirm long-term impacts aligned with our original hypothesis. Temporal drivers linked to the hydrological cycle explained most of the variance in limnological parameters and trace elements, underscoring the dominant role of seasonal and interannual rainfall patterns in structuring water quality dynamics in this tropical system. These patterns were markedly amplified in the Doce River sub-basin, indicating altered hydro-sedimentological functioning and continued mobilization of legacy contaminants during rainy periods.

Spatial patterns further revealed that the most pronounced effects persist upstream of the Santo Antônio confluence, where tailings influence remains strongest, while downstream regions show partial attenuation yet still elevated concentrations of key parameters. In contrast, the Santo Antônio River displayed a more stable and typical seasonal regime, reinforcing its role as an unimpacted reference system. Together, these findings provide clear evidence that both baseline conditions and seasonal trajectories remain altered in the impacted basin. This highlights the need for continued long-term and spatially explicit monitoring to capture subtle but ecologically meaningful changes, support recovery assessments, and inform watershed-scale management strategies aimed at mitigating the enduring environmental legacy of the disaster.

## Supplementary Information

Below is the link to the electronic supplementary material.

ESM 1 (DOCX 161 KB)

## Data Availability

The authors declare that the data supporting the findings of this study are available within the paper and its Supplementary Information file. Should any raw data files be needed in another format they are available from the corresponding author upon reasonable request.

## Declarations

All authors have read, understood, and have complied as applicable with the statement on “Ethical responsibilities of Authors” as found in the Instructions for Authors.”
