# Supplementary material for: A decade on: the tailings legacy in Rio Doce water quality
Source: Environ Monit Assess. 2026 Jun 20;198(7):751. doi: 10.1007/s10661-026-15549-x (PMC13283196; doi:10.1007/s10661-026-15549-x)
Supplement: Supplementary file 1 — (DOCX 161 KB) [file 10661_2026_15549_MOESM1_ESM.docx]

**Supplementary material**

**A Decade On: The Tailings Legacy in Rio Doce Water Quality**

Luciana Pena Mello Brandão¹*, Bianca Loureiro do Valle¹, Renata Cristina Henedino Amancio¹, Winnícius Muniz dos Santos Sá¹, Arielli Giachini Zavaski¹, Estevão Emerick de Oliveira Eller ¹, Leticia Malta Costa², José Fernandes Bezerra-Neto¹

¹Limnea—Institute of Biological Sciences (ICB), Federal University of Minas Gerais (UFMG), Av. Antônio Carlos 6627, Pampulha, 31270-901, Belo Horizonte, Minas Gerais, Brazil

² Departament of Chemistry, ICEX, Federal University of Minas Gerais (UFMG), Av. Antônio Carlos 6627, Pampulha, 31270-901, Belo Horizonte, Minas Gerais, Brazil


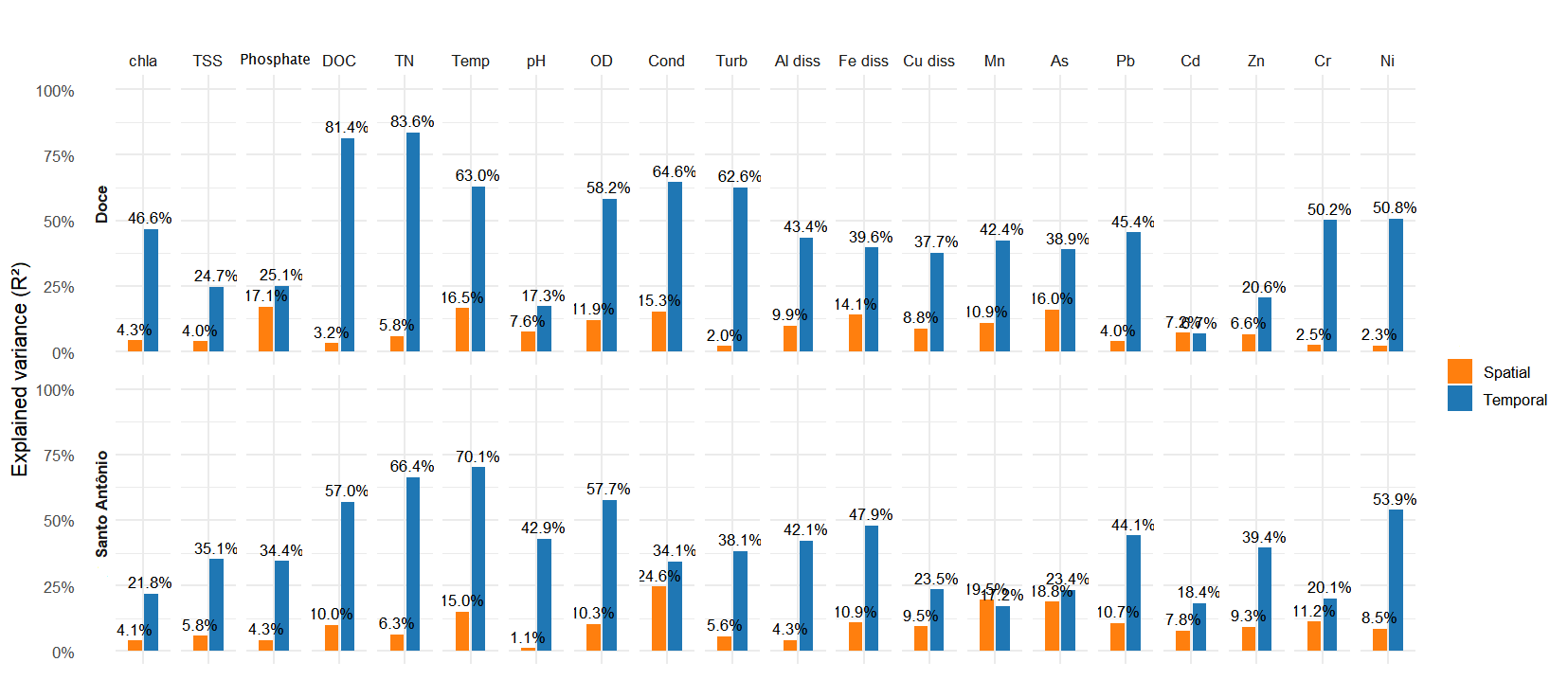


Figure S1 - Variance partitioning of individual environmental variables by sampling campaign (temporal) and sampling point (spatial) for each sub-basin. Bars represent the proportion of variance explained (R²) by each factor.


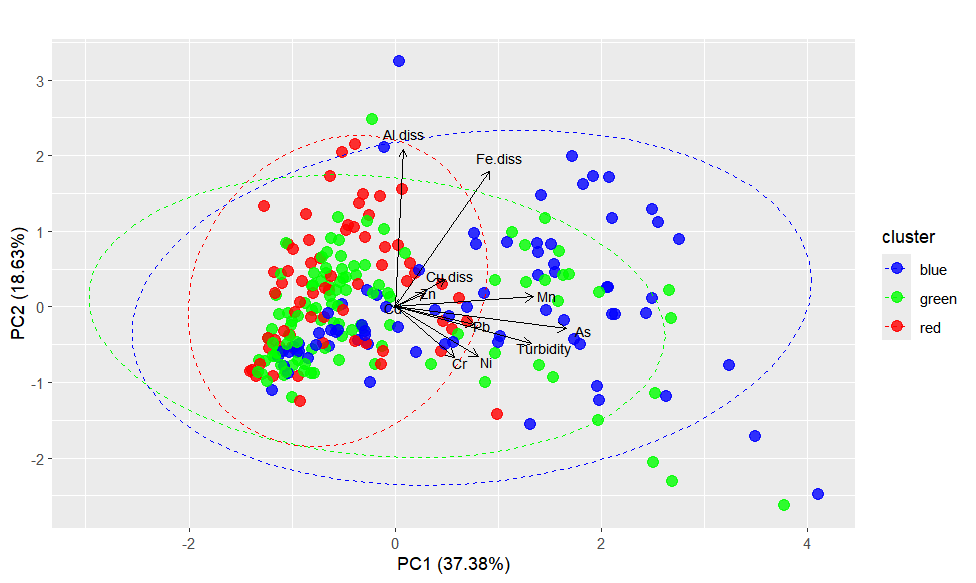


Figure S2 - Principal Component Analysis (PCA) of standardized variables. A) Scores with 95% confidence ellipses by groups defined in the cluster analysis. (blue = Upstream Doce River; Green = Downstream Doce River; Red = Santo Antônio River). Variables used: turbidity (Turb; NTU), dissolved aluminum (Al diss; µg L⁻¹), dissolved iron (Fe diss; µg L⁻¹), dissolved copper (Cu diss; µg L⁻¹), and total metals Mn, As, Pb, Cd, Zn, Cr, Ni (µg L⁻¹). Loadings are shown as vectors; axes display variance explained (%).


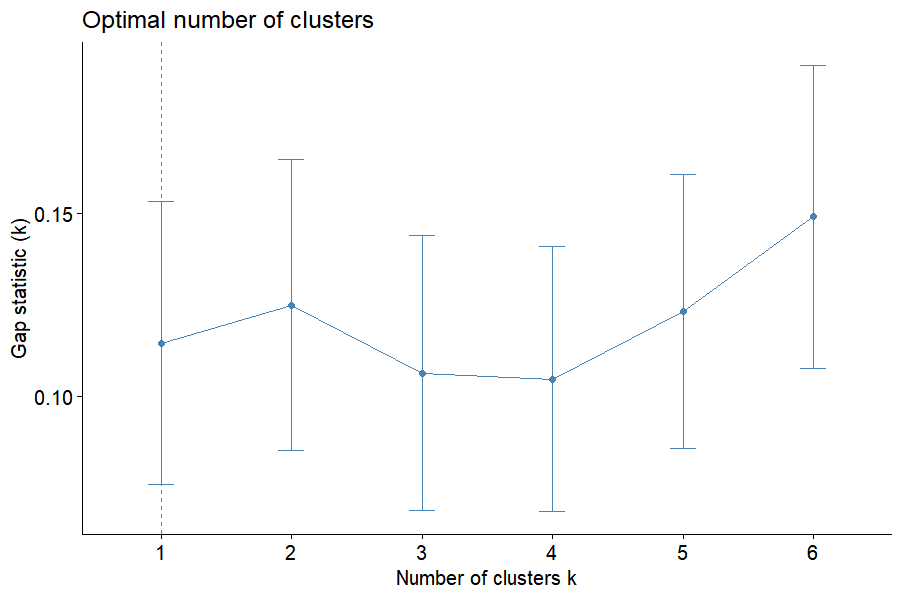


Figure S3 - Evaluation of the optimal number of clusters based on the average silhouette width, Calinski–Harabasz index, and gap statistic, considering solutions from k = 2 to k = 6.

Tabela S1 - Mean and standard deviation of limnological variables and metal concentrations by sampling site.

|  |  | **River Doce sites** | | | | | | | | | | | | | | **River Santo Antônio sites** | | | | | |
| --- | --- | --- | --- | --- | --- | --- | --- | --- | --- | --- | --- | --- | --- | --- | --- | --- | --- | --- | --- | --- | --- |
| Parameters |  | **10** | **11A** | **12A** | **13** | **15A** | **1B** | **2J** | **3A** | **4A** | **5** | **6A** | **7A** | **8A** | **9A** | **16** | **17A** | **18A** | **18C** | **19A** | **21** |
| Chlorophyll-a | Mean | 0.27 | 5.30 | 1.15 | 0.48 | 1.05 | 2.62 | 1.71 | 1.05 | 1.38 | 2.45 | 1.91 | 5.33 | 5.03 | 4.97 | 2.02 | 0.72 | 3.62 | 1.27 | 2.47 | 5.94 |
|  | SD | 0.77 | 15.18 | 4.46 | 0.94 | 2.60 | 10.09 | 4.56 | 3.53 | 4.07 | 5.53 | 4.99 | 14.91 | 17.18 | 15.79 | 3.77 | 1.79 | 17.03 | 3.83 | 3.41 | 16.99 |
| TSS | Mean | 150.93 | 146.53 | 172.72 | 212.35 | 143.26 | 158.23 | 39.19 | 151.88 | 279.36 | 195.23 | 144.65 | 125.01 | 118.92 | 106.92 | 66.60 | 78.21 | 134.89 | 36.81 | 161.30 | 135.26 |
|  | SD | 228.75 | 201.37 | 279.20 | 433.14 | 176.13 | 221.07 | 40.95 | 274.00 | 523.05 | 245.57 | 208.82 | 128.56 | 142.49 | 157.61 | 107.92 | 94.13 | 287.58 | 38.10 | 269.51 | 186.70 |
| Phosphate | Mean | 15.86 | 10.73 | 13.85 | 5.73 | 19.44 | 35.36 | 23.58 | 18.01 | 6.04 | 2.09 | 54.15 | 22.80 | 16.57 | 5.72 | 31.33 | 27.71 | 27.30 | 60.81 | 27.66 | 11.34 |
|  | SD | 13.40 | 10.59 | 18.02 | 8.35 | 52.17 | 78.89 | 32.54 | 62.67 | 16.99 | 5.62 | 95.89 | 20.94 | 24.43 | 7.95 | 46.17 | 42.78 | 74.94 | 147.70 | 44.14 | 21.20 |
| DOC | Mean | 5.96 | 7.19 | 6.38 | 6.51 | 6.99 | 6.06 | 4.21 | 5.87 | 5.73 | 6.47 | 5.98 | 8.24 | 7.40 | 7.37 | 8.48 | 10.65 | 8.41 | 12.11 | 17.85 | 8.82 |
|  | SD | 3.08 | 4.31 | 4.12 | 4.58 | 6.55 | 4.62 | 1.54 | 4.83 | 4.61 | 6.29 | 4.72 | 5.93 | 5.57 | 8.49 | 6.08 | 17.36 | 5.82 | 11.77 | 18.77 | 5.85 |
| TN | Mean | 2.60 | 2.67 | 2.30 | 2.30 | 2.02 | 1.77 | 0.61 | 1.36 | 2.31 | 2.31 | 1.52 | 2.54 | 3.12 | 2.53 | 1.42 | 1.31 | 1.22 | 0.34 | 1.28 | 0.88 |
|  | SD | 2.84 | 2.57 | 2.30 | 2.27 | 2.16 | 1.75 | 0.22 | 1.71 | 2.64 | 2.42 | 1.65 | 2.98 | 3.27 | 2.76 | 1.46 | 1.43 | 1.42 | 0.28 | 2.22 | 0.75 |
| Temperature | Mean | 27.57 | 26.71 | 27.73 | 27.15 | 27.27 | 25.40 | 25.69 | 26.16 | 26.25 | 25.86 | 26.09 | 26.05 | 27.40 | 26.74 | 23.10 | 23.96 | 24.67 | 23.96 | 25.22 | 25.59 |
|  | SD | 1.68 | 2.28 | 1.44 | 1.52 | 1.93 | 2.68 | 2.97 | 2.78 | 2.47 | 2.49 | 2.33 | 2.13 | 1.72 | 1.58 | 3.03 | 2.08 | 1.87 | 2.01 | 1.70 | 2.30 |
| pH | Mean | 7.37 | 7.25 | 7.50 | 7.46 | 7.19 | 7.34 | 7.76 | 7.24 | 7.24 | 7.20 | 7.13 | 7.15 | 7.31 | 7.33 | 7.29 | 7.31 | 7.32 | 7.15 | 7.14 | 7.13 |
|  | SD | 0.60 | 0.33 | 0.33 | 0.35 | 0.57 | 0.46 | 1.17 | 0.38 | 0.37 | 0.41 | 0.33 | 0.27 | 0.30 | 0.28 | 1.10 | 0.47 | 0.59 | 0.60 | 0.61 | 0.39 |
| OD | Mean | 8.79 | 8.52 | 8.93 | 8.75 | 9.46 | 8.98 | 8.53 | 8.73 | 8.56 | 8.79 | 9.04 | 8.76 | 8.79 | 8.63 | 9.30 | 9.35 | 9.18 | 9.37 | 8.92 | 8.92 |
|  | SD | 0.86 | 0.31 | 0.74 | 0.51 | 0.78 | 0.66 | 0.60 | 0.63 | 0.91 | 0.87 | 0.60 | 0.84 | 0.39 | 0.64 | 1.00 | 0.57 | 0.64 | 0.64 | 0.98 | 0.38 |
| Conductivity | Mean | 90.62 | 88.72 | 88.61 | 87.74 | 101.06 | 63.98 | 60.50 | 71.97 | 70.65 | 70.13 | 71.29 | 97.17 | 88.88 | 85.84 | 40.62 | 42.16 | 41.63 | 27.99 | 89.43 | 46.83 |
|  | SD | 32.00 | 37.84 | 29.96 | 29.88 | 31.82 | 16.58 | 17.63 | 36.84 | 19.94 | 19.66 | 24.00 | 38.68 | 28.32 | 31.16 | 17.28 | 19.13 | 22.19 | 17.97 | 101.68 | 23.54 |
| Turbidity | Mean | 127.58 | 146.97 | 150.71 | 190.30 | 74.27 | 121.20 | 185.65 | 138.51 | 149.86 | 132.14 | 128.08 | 136.38 | 105.62 | 118.96 | 117.85 | 151.43 | 49.15 | 42.11 | 98.07 | 133.51 |
|  | SD | 222.32 | 227.52 | 242.98 | 284.82 | 90.32 | 163.68 | 186.15 | 180.63 | 124.70 | 104.51 | 108.58 | 145.76 | 187.70 | 191.25 | 197.41 | 247.51 | 60.23 | 47.86 | 222.09 | 239.51 |
| Al diss | Mean | 46.48 | 42.72 | 31.22 | 31.21 | 62.18 | 23.79 | 70.71 | 30.18 | 23.45 | 19.58 | 19.87 | 53.90 | 33.80 | 37.59 | 39.32 | 46.32 | 54.11 | 30.78 | 20.75 | 45.22 |
|  | SD | 57.00 | 39.33 | 46.02 | 38.56 | 86.28 | 47.73 | 121.49 | 37.66 | 32.03 | 24.24 | 27.65 | 112.72 | 38.30 | 46.97 | 57.02 | 48.49 | 110.60 | 37.35 | 37.73 | 89.01 |
| Fe diss | Mean | 204.01 | 204.73 | 192.76 | 168.11 | 156.81 | 303.88 | 390.39 | 304.38 | 307.92 | 251.48 | 228.67 | 295.54 | 193.78 | 204.77 | 274.87 | 283.63 | 259.35 | 289.41 | 223.12 | 195.69 |
|  | SD | 127.71 | 121.00 | 162.74 | 116.66 | 140.35 | 154.07 | 320.80 | 186.39 | 178.83 | 107.84 | 83.02 | 423.01 | 128.68 | 116.59 | 142.48 | 101.51 | 84.12 | 129.18 | 108.63 | 95.36 |
| Cu diss | Mean | 0.26 | 0.34 | 0.40 | 0.30 | 0.51 | 1.08 | 0.37 | 1.53 | 0.73 | 0.86 | 1.13 | 1.35 | 0.91 | 0.75 | 0.39 | 2.37 | 0.78 | 0.30 | 0.47 | 0.74 |
|  | SD | 0.54 | 0.74 | 0.59 | 0.62 | 1.39 | 1.79 | 0.64 | 4.44 | 1.35 | 1.52 | 1.70 | 3.61 | 1.25 | 1.04 | 0.86 | 7.80 | 1.35 | 0.56 | 0.83 | 1.65 |
| Mn | Mean | 114.75 | 121.49 | 129.53 | 135.41 | 36.14 | 174.49 | 99.49 | 194.09 | 170.25 | 172.34 | 196.30 | 192.06 | 65.52 | 104.04 | 31.69 | 50.70 | 52.53 | 64.75 | 415.19 | 94.52 |
|  | SD | 154.01 | 152.07 | 193.00 | 186.69 | 64.28 | 160.63 | 96.22 | 185.92 | 109.73 | 130.04 | 148.56 | 188.18 | 55.89 | 110.16 | 23.81 | 37.52 | 33.27 | 42.69 | 689.21 | 64.35 |
| As | Mean | 1.14 | 1.26 | 0.98 | 1.01 | 0.45 | 2.50 | 1.36 | 3.14 | 2.38 | 1.88 | 2.42 | 1.87 | 0.92 | 1.12 | 0.03 | 0.19 | 0.19 | 0.25 | 0.22 | 0.57 |
|  | SD | 1.72 | 1.54 | 1.19 | 1.37 | 0.53 | 2.39 | 1.07 | 3.94 | 1.98 | 1.65 | 2.19 | 1.84 | 1.00 | 1.22 | 0.13 | 0.26 | 0.25 | 0.32 | 0.51 | 0.71 |
| Pb | Mean | 4.23 | 5.49 | 4.26 | 4.78 | 1.69 | 4.95 | 2.55 | 3.11 | 2.65 | 2.76 | 2.93 | 3.72 | 3.43 | 6.72 | 0.66 | 1.25 | 1.99 | 1.25 | 5.55 | 7.27 |
|  | SD | 7.35 | 8.05 | 7.46 | 8.54 | 3.12 | 7.06 | 3.13 | 4.86 | 2.56 | 2.78 | 3.46 | 4.21 | 5.22 | 16.69 | 0.80 | 1.78 | 2.43 | 1.56 | 13.10 | 11.27 |
| Cd | Mean | 0.00 | 0.02 | 0.02 | 0.00 | 0.00 | 0.01 | 0.00 | 0.00 | 0.00 | 0.05 | 0.01 | 0.00 | 0.00 | 0.00 | 0.00 | 0.01 | 0.00 | 0.00 | 0.03 | 0.13 |
|  | SD | 0.01 | 0.10 | 0.07 | 0.02 | 0.00 | 0.02 | 0.00 | 0.02 | 0.00 | 0.16 | 0.04 | 0.00 | 0.00 | 0.00 | 0.00 | 0.03 | 0.02 | 0.00 | 0.07 | 0.45 |
| Zn | Mean | 12.14 | 14.94 | 13.83 | 15.87 | 7.31 | 10.83 | 7.66 | 7.46 | 6.99 | 3.45 | 3.67 | 9.43 | 7.44 | 14.63 | 4.18 | 11.48 | 19.32 | 4.96 | 17.08 | 17.74 |
|  | SD | 16.35 | 19.28 | 27.62 | 29.93 | 21.23 | 18.11 | 12.36 | 13.44 | 11.27 | 10.01 | 8.49 | 17.87 | 17.90 | 31.08 | 9.22 | 28.39 | 38.87 | 12.48 | 29.54 | 24.20 |
| Cr | Mean | 5.39 | 6.45 | 7.28 | 8.90 | 1.31 | 10.43 | 3.40 | 7.30 | 6.32 | 4.41 | 5.86 | 5.92 | 9.10 | 7.24 | 0.51 | 1.83 | 2.61 | 0.99 | 3.85 | 10.12 |
|  | SD | 14.22 | 14.36 | 18.77 | 22.33 | 2.08 | 19.84 | 7.10 | 15.93 | 11.04 | 6.80 | 9.45 | 10.82 | 29.55 | 16.45 | 1.01 | 3.42 | 4.39 | 1.88 | 7.30 | 22.58 |
| Ni | Mean | 11.85 | 13.57 | 15.23 | 17.68 | 3.55 | 15.76 | 6.11 | 14.11 | 11.24 | 8.22 | 10.68 | 10.71 | 8.78 | 12.81 | 0.87 | 3.25 | 5.01 | 1.45 | 9.84 | 13.48 |
|  | SD | 28.89 | 28.91 | 35.83 | 41.26 | 7.54 | 31.58 | 10.38 | 33.10 | 18.52 | 12.56 | 17.93 | 19.79 | 19.59 | 24.31 | 2.35 | 7.36 | 8.54 | 3.69 | 23.55 | 24.18 |

Table S2 - Descriptive statistics for each cluster group.

| Cluster_Group | Parameter | mean | SD | min | max |
| --- | --- | --- | --- | --- | --- |
| Blue | Al diss | 31.27 | 19.70 | 19.58 | 70.71 |
| Green | Al diss | 38.10 | 10.04 | 20.75 | 53.90 |
| Red | Al diss | 46.54 | 12.27 | 30.78 | 62.18 |
| Blue | As | 2.28 | 0.61 | 1.36 | 3.14 |
| Green | As | 1.01 | 0.45 | 0.22 | 1.87 |
| Red | As | 0.22 | 0.15 | 0.03 | 0.45 |
| Blue | Cd | 0.01 | 0.02 | 0.00 | 0.05 |
| Green | Cd | 0.02 | 0.04 | 0.00 | 0.13 |
| Red | Cd | 0.00 | 0.00 | 0.00 | 0.01 |
| Blue | Chla | 1.85 | 0.60 | 1.05 | 2.62 |
| Green | Chla | 3.44 | 2.32 | 0.27 | 5.94 |
| Red | Chla | 1.74 | 1.16 | 0.72 | 3.62 |
| Blue | Conductivity | 68.09 | 4.70 | 60.50 | 71.97 |
| Green | Conductivity | 84.87 | 14.61 | 46.83 | 97.17 |
| Red | Conductivity | 50.69 | 28.76 | 27.99 | 101.06 |
| Blue | Cr | 6.29 | 2.46 | 3.40 | 10.43 |
| Green | Cr | 7.14 | 1.99 | 3.85 | 10.12 |
| Red | Cr | 1.45 | 0.81 | 0.51 | 2.61 |
| Blue | Cu diss | 0.95 | 0.39 | 0.37 | 1.53 |
| Green | Cu diss | 0.61 | 0.36 | 0.26 | 1.35 |
| Red | Cu diss | 0.87 | 0.86 | 0.30 | 2.37 |
| Blue | DOC | 5.72 | 0.78 | 4.21 | 6.47 |
| Green | DOC | 8.41 | 3.65 | 5.96 | 17.85 |
| Red | DOC | 9.33 | 2.03 | 6.99 | 12.11 |
| Blue | Fe diss | 297.78 | 56.00 | 228.67 | 390.39 |
| Green | Fe diss | 209.17 | 35.52 | 168.11 | 295.54 |
| Red | Fe diss | 252.82 | 54.85 | 156.81 | 289.41 |
| Blue | Mn | 167.83 | 35.33 | 99.49 | 196.30 |
| Green | Mn | 152.50 | 104.31 | 65.52 | 415.19 |
| Red | Mn | 47.16 | 13.34 | 31.69 | 64.75 |
| Blue | Ni | 11.02 | 3.58 | 6.11 | 15.76 |
| Green | Ni | 12.66 | 2.76 | 8.78 | 17.68 |
| Red | Ni | 2.82 | 1.67 | 0.87 | 5.01 |
| Blue | OD | 8.77 | 0.21 | 8.53 | 9.04 |
| Green | OD | 8.78 | 0.14 | 8.52 | 8.93 |
| Red | OD | 9.33 | 0.10 | 9.18 | 9.46 |
| Blue | Pb | 3.16 | 0.90 | 2.55 | 4.95 |
| Green | Pb | 5.05 | 1.32 | 3.43 | 7.27 |
| Red | Pb | 1.37 | 0.50 | 0.66 | 1.99 |
| Blue | TN | 1.65 | 0.64 | 0.61 | 2.31 |
| Green | TN | 2.25 | 0.71 | 0.88 | 3.12 |
| Red | TN | 1.26 | 0.60 | 0.34 | 2.02 |
| Blue | TSS | 161.42 | 77.94 | 39.19 | 279.36 |
| Green | TSS | 147.77 | 31.95 | 106.92 | 212.35 |
| Red | TSS | 91.96 | 45.69 | 36.81 | 143.26 |
| Blue | Temperature | 25.91 | 0.32 | 25.40 | 26.25 |
| Green | Temperature | 26.68 | 0.89 | 25.22 | 27.73 |
| Red | Temperature | 24.59 | 1.60 | 23.10 | 27.27 |
| Blue | Turbidity | 142.57 | 23.24 | 121.20 | 185.65 |
| Green | Turbidity | 134.23 | 27.36 | 98.07 | 190.30 |
| Red | Turbidity | 86.96 | 46.67 | 42.11 | 151.43 |
| Blue | Zn | 6.68 | 2.77 | 3.45 | 10.83 |
| Green | Zn | 13.68 | 3.44 | 7.44 | 17.74 |
| Red | Zn | 9.45 | 6.21 | 4.18 | 19.32 |
| Blue | pH | 7.32 | 0.23 | 7.13 | 7.76 |
| Green | pH | 7.29 | 0.14 | 7.13 | 7.50 |
| Red | pH | 7.25 | 0.08 | 7.15 | 7.32 |
| Blue | phosphate | 23.20 | 19.36 | 2.09 | 54.15 |
| Green | phosphate | 14.47 | 7.31 | 5.72 | 27.66 |
| Red | phosphate | 33.32 | 15.97 | 19.44 | 60.81 |
